# Molecular Detection of Persistent *Francisella tularensis* Subspecies *holarctica* in Natural Waters

**DOI:** 10.1155/2011/851946

**Published:** 2010-09-08

**Authors:** T. Broman, J. Thelaus, A.-C. Andersson, S. Bäckman, P. Wikström, E. Larsson, M. Granberg, L. Karlsson, E. Bäck, H. Eliasson, R. Mattsson, A. Sjöstedt, M. Forsman

**Affiliations:** ^1^Department of CBRN Defence and Security, Swedish Defence Research Agency, 901 82 Umeå, Sweden; ^2^Department of Infectious Diseases, Örebro University Hospital, 701 85 Örebro, Sweden; ^3^National Veterinary Institute, 751 89 Uppsala, Sweden; ^4^Department of Clinical Microbiology, Umeå University, 901 87 Umeå, Sweden

## Abstract

Tularemia, caused by the bacterium *Francisella tularensis*, where *F. tularensis* subspecies *holarctica* has long been the cause of endemic disease in parts of northern Sweden. Despite this, our understanding of the natural life-cycle of the organism is still limited. During three years, we collected surface water samples (*n* = 341) and sediment samples (*n* = 245) in two areas in Sweden with endemic tularemia. Real-time PCR screening demonstrated the presence of *F. tularenis lpnA* sequences in 108 (32%) and 48 (20%) of the samples, respectively. The 16S rRNA sequences from those samples all grouped to the species *F. tularensis*. Analysis of the FtM19InDel region of *lpnA*-positive samples from selected sampling points confirmed the presence of *F. tularensis* subspecies *holarctica*-specific sequences. These sequences were detected in water sampled during both outbreak and nonoutbreak years. Our results indicate that diverse *F. tularensis*-like organisms, including *F. tularensis* subsp. *holarctica*, persist in natural waters and sediments in the investigated areas with endemic tularemia.

## 1. Introduction

Tularemia is a zoonotic disease caused by the bacterium *Francisella tularensis*. At present, four subspecies of *F. tularensis* are suggested [1, 2], two of which are of clinical importance (subsp. *tularensis* and *holarctica*, [2]). *Francisella tularensis* subsp. *tularensis* strains only occur in North America [3, 4] whereas *F. tularensis* subsp. *holarctica* strains are found throughout the Northern Hemisphere [5]. *Francisella tularensis* is categorized as a category A potential bioterrorism agent. Recently, it was established that diverse *Francisella*-like bacteria exist in the environment (in soil, seawater, and fish) [6–10]. These *Francisella*-like organisms cluster in various genetic clades together with tick endosymbionts, fish pathogens, and bacteria detected in soil and sediment [5].

The epizootiology of *F. tularensis* is complex, involving numerous wildlife species and several potential vectors for its transmission as a disease-causing agent. Indeed, tularemia has been detected in approximately 250 wildlife species, giving *F. tularensis* a broader host range than any other known zoonotic disease-causing organism [11]. Various bloodsucking arthropods have been found naturally infected with the bacterium, like ticks, tabanid flies, midges, mites, fleas, lice, and mosquitoes [12]. Nevertheless, local tularemia outbreaks are often patchy, occurring around natural foci in geographically restricted areas, typically in association with just one or a few key mammalian and arthropod species.

Tularemia (caused by *F. tularensis* subsp. *holarctica* strains) is endemic in areas of northern Sweden and, during the past decade, has emerged in areas of central Sweden too. In these areas, it is a local public health threat since it occurs at a high frequency, especially in late summer and autumn. The reasons for its geographical distribution and seasonal occurrence are unknown. It is generally thought that naturally infected mosquitoes are the major transmission vectors of tularemia in Sweden [13], with the occurrence of naturally infected *Aedes cinereus *reported as early as 1942 [14]. It is still not clear how mosquito vectors acquire the bacteria.

*Francisella tularensis* subsp. *holarctica* is often associated with water environments like streams, ponds, lakes, and rivers [15, 16]. Water-borne transmission of tularemia (subsp. *holarctica*) has been frequently reported [17–25]. The presence of *F. tularensis* in water and sediments has been proven by its isolation from laboratory animals inoculated with samples [21]. However, the role of natural waters in the long-term survival of clinically relevant subspecies is not well characterized, as it has not been possible to directly culture the bacteria from water samples. However, experiments have shown that *F. tularensis* subsp. *holarctica* survive in watercourses, possibly in association with protozoa [26–29]. The larvae of flood-water mosquitoes significantly prey on the protozoan community [30], and may well be exposed to *F. tularensis* subsp. *holarctica* in this way.

In the study presented here, we used molecular detection techniques to confirm the persistence of *F. tularensis* subsp. *holarctica* DNA in natural surface waters over a three-year period in two Swedish tularemia regions. Water, sediments, and small rodents were sampled in two regions with some of the highest incidences of tularemia reported in Sweden.

## 2. Materials and Methods

### 2.1. Study Regions

**Figure 1:**
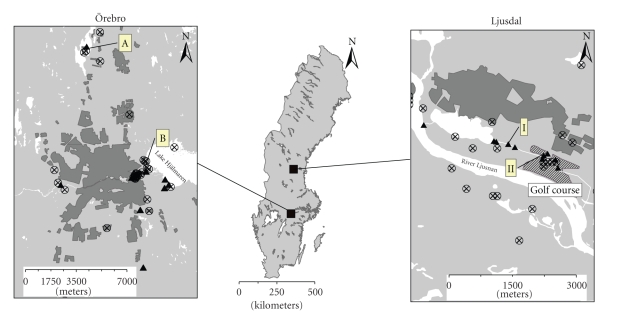
Sampling locations (black triangles), in the Ljusdal and the Örebro area. Roman numerals (I, II) and letters (A, B) indicate sampling points selected for detailed analysis. Waterways are represented in white, urban areas are shaded. Each encircled × shows the probable place of disease transmission for patients infected in 1998–2005 (Ljusdal) and 2003-2004 (Örebro) [31].

The study was conducted in two regions with reoccurring tularemia in Sweden: Ljusdal and Örebro. The municipality of Ljusdal (61° 50′0′′ N 16°5′0′′ E), with a population of 19 384 (2005), situated in the county of Gävleborg (a population of 275 994, 2005), has a history of tularemia outbreaks dating back to at least the 1930s. This region is typical of endemic tularemia regions, in which outbreaks occur in geographically restricted areas at irregular intervals. Since 1931, at least 2500 human cases have been recorded in the county. Data indicate that most patients have acquired the infection within or close to the Ljusdal municipality or on a nearby golf course (Figure 1) [31].

In recent years, the disease has emerged in Örebro county (59° 16′0′′ N 15° 12′0′′ E), located 364 km south of Ljusdal, with a population of 274 121 (2005). Before 2000 only a handful of cases were reported from the county and limited numbers of cases occurred in 2001 and 2002. However, between 2003 and 2005, 229 human cases of tularemia were reported (http://www.smi.se/in-english/statistics/tularaemia/) (Table 1). The tularemia cases have clustered in distinct areas, namely: (i) along the west shores of Lake Hjälmaren, (ii) close to the city center along River Svartån, (iii) in an area with allotment gardens close to the city center, and (iv) around Lake Lången (Figure 1) [31].

For the first year sampling (2003), several sampling points were chosen (26 in Ljusdal and 21 in Örebro), based on the knowledge of local physicians about the geographical distribution of human tularemia cases (Figure 1). In 2004 and 2005, there were ten sampling points in each study region.

### 2.2. Small Rodents

Rodents were collected using live-traps (under ethical permit number C 118/3 issued by the Local Ethical Committee on Laboratory Animals in Umeå, Sweden), baited with a mixture of carrots, potatoes, oatmeal, and pieces of apples, from mid-May to mid-September. Trapping was performed in Ljusdal (on four occasions) and Örebro (two occasions) during 2003, and in Örebro (five occasions) during 2004. At each sampling during 2003, traps were set for five days, and in 2004 traps were set for two to five days. Traps were checked every 12 hours. Trapped rodents were anesthetized using halothane and euthanized through cervical dislocation. Carcasses were kept refrigerated during transportation to a local laboratory, where spleen and liver samples were prepared and deep-frozen (within four hours of euthanization) until further analysis. After thawing, spleen and liver samples were used for *F. tularensis* cultivation and DNA preparation for polymerase chain reaction (PCR) analysis.

*Francisella tularensis* was cultured on modified Thayer-Martin agar plates [32] at 37°C in 5% CO_2_ for six days, and its growth was confirmed by slide agglutination with a commercial antiserum (Difco Laboratories, Augsburg, Germany). DNA was purified using the guanidine isothiocyanate method [33]. This was followed by real-time PCR probe-based *lpnA* assays [27] and typing with multiple locus variable-number tandem repeat analysis (MLVA) [3].

### 2.3. Water and Sediment Samples

Samples were collected on several occasions during summer, from mid-May to mid-September, during three consecutive years (on four, seven and three occasions in Ljusdal and on two, eight and three occasions in Örebro, during the years 2003, 2004, and 2005, resp.) (Figure 1). Samples were collected from both surface water and sediment. In 2003, sediment samples were collected from two of the sampling points in Ljusdal and from all of the sampling points in Örebro. In 2004 and 2005, sediments were sampled from all sampling points. The samples were collected as single-grab samples in 100 ml plastic tubes. They were refrigerated during transportation to the laboratory (within 24 and 48 hours for Örebro and Ljusdal samples, resp.). DNA extraction was performed upon arrival and the purified DNA was stored at −20°C until further analysis.

### 2.4. DNA Purification and PCR Analysis of Water and Sediment

Two mL of each water or sediment sample was centrifuged at 16 000 × g for 1 hour, 1.9 mL of the resulting supernatant was discarded and DNA was extracted from the remaining volume using a SoilMaster DNA Extraction Kit according to the recommendations of the manufacturer for environmental water samples (Epicentre Biotechnologies, Madison, WI, USA). To increase the yield of DNA the samples were incubated at 37°C for ten minutes, without shaking, after Proteinase K treatment. The resulting DNA pellet was resuspended in 60 *μ*L of TE buffer and either frozen and stored or immediately subjected to PCR analysis. As negative controls, 2 mL samples of sterile water were treated according to the protocol described above. Sample preparation, PCR reaction preparation and thermal cycling were separated and performed in different rooms.

Water and sediment samples were screened for *F. tularensis* using a real-time PCR probe-based assay (iQFt1F/R) for detection of the *F. tularensis*-specific *lpnA* sequence, as previously described [27]. To detect false negative results caused by PCR inhibitory substances, the assay also included an internal control probe [27]. All samples were analyzed in at least triplicate PCR reactions. Samples from selected sampling points (described below) were further subjected to a *F. tularensis* subsp. *holarctica*-specific-PCR based on the 30 bp-deletion region FtM19 [3, 4, 34–36], followed by fragment size analysis [34]. Each reaction consisted of 1 *μ*L template, 1x Amplitaq GOLD PCR buffer, 40 *μ*M each of the primers FtM19InDelF/R (WELLRED 5′-CCAGTACAAACTCAATT TGGTTATCATC-3′ and 5′-GTTTCAGAATTCATTTTTGTCCGTAA-3′), 2.6 mM MgCl_2_, 1 M betaine, 0.2 mM dNTP, 0.5 U Amplitaq GOLD polymerase, and MilliQ water to give a total volume of 12.5 *μ*L. An initial denaturation at 94°C for 2 minutes was followed by 50 cycles of 94°C for 30 seconds, 60°C for 30 s and 72°C for 30 seconds, followed by final incubation at 72°C for 5 minutes in a MyCycler thermal cycler (Bio-Rad Laboratories, Hercules, CA). Positive control mixtures using DNA from *F. tularensis* subsp. *holarctica,* and negative control mixtures without a template, were included in each PCR run. The resulting amplicons were sized by capillary electrophoresis using a CEQ 8800 Genetic Analysis System (Beckman Coulter Inc., Fullerton, CA, USA) after mixing 1 *μ*L of the PCR products from each amplification with standards (from a CEQ DNA size standard kit-400) in sample loading solution according to the manufacturer's manual.

### 2.5. Sequencing

The *lpnA* and FtM19InDel PCR amplicons were purified using MicroSpin S-400 HR columns (GE Healthcare Bio-Sciences, Uppsala, Sweden), then sequenced using a CEQ8800 Genetic Analysis System and a DTCS Quick Start kit (Beckman Coulter Inc. Fullerton, CA, USA) according to the manufacturer's instructions, with iQFt1F/R and FtM19InDelF/R primers, respectively. Acquired sequences were deposited with GenBank under accession numbers FJ94649, FJ946492 to FJ946499 *(lpnA*) and FJ946500 to FJ946512 (FtM19InDel).

### 2.6. 16S rRNA Cloning and Sequencing

Amplification, direct cloning and subsequent sequencing of 16S rRNA was performed on samples chosen for detailed studies. 16S rRNA *Francisella*-specific primers Fr153F0.1 (5′-GCCCATTTGAGGGGGATACC-3′) and Fr1281R0.1 (5′-GGACTAAGAGTACCTTTTTGAGT-3′) were used as previously described [6]. 16S rRNA PCR products were purified on SeaKem agarose gels (Cambrex North Brunswick, Inc., North Brunswick, NJ, USA) and excised bands were eluted using GenElute Gel Spin Columns (Sigma-Aldrich, St. Louis, MO, USA). Products were cloned into the pCRII vector using a TOPO-TA Cloning Kit according to the protocol recommended by the manufacturer (Invitrogen Co., Carlsbad, CA, USA). Fifty clones, representing each PCR reaction, were subsequently picked and stored in glycerol at −70°C prior to sequencing. Plasmid DNA was isolated from overnight cultures using an E.Z.N.A. Plasmid Miniprep Kit (Omega Bio-Tek Inc., Doraville, GA, USA) and sequenced using universal M13F and M13R primers. Detected sequences (~1150 bp) were deposited with GenBank under accession numbers DQ994171 to DQ994200.

### 2.7. Phylogenetic Analysis of Sequence Data

To evaluate the sequence similarity of the *Francisella* sequences obtained, reference sequences from GenBank were included in ClustalW alignment, performed using MEGA version 3.1 [37]. For the 16S rRNA sequences, a phylogenetic tree was generated using maximum parsimony analysis and bootstrapping.

## 3. Results

### 3.1. Human Cases

During the three-year study period, 19 human tularemia cases were verified in the Ljusdal area (County Medical Officer, Gävle-Sandviken, Personal communication) and 229 in Örebro county (Table 1).

### 3.2. Small Rodents

During the first year of the study 97 rodents (60 in Ljusdal and 37 in Örebro, Table 1) were caught alive. The presence of *F. tularensis* in spleen and liver samples of the rodents was investigated by culture and PCR analysis. Two rodents, a water vole (*Arvicola terrestris*) and a yellow-necked mouse (*Apodemus flavicollis*) were infected with *F. tularensis, *as demonstrated by the culture assays and PCR analysis. Both were from the Örebro area, but from different sampling points. Genotyping identified the isolates as *F. tularensis *subsp*. holarctica. *Subtyping by MLVA showed that the isolates were distinct, and thus likely contracted from different sources (data not shown). The rodent population declined in 2004 and despite intensified sampling only seven individuals was trapped in Örebro, all of which were *Francisella*-negative (Table 1).

### 3.3. Francisella tularensis in Water and Sediment Samples

**Figure 2:**
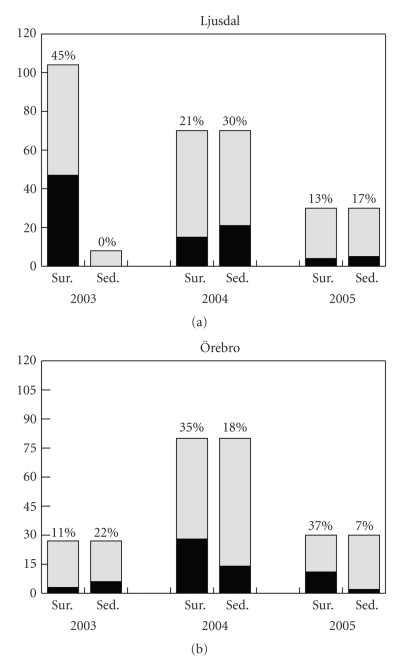
Stacked bar graph showing numbers of positive (black) and negative (light grey) samples during the sampling period (2003–2005) in Ljusdal (a) and Örebro (b). The percentages of samples positive for *F. tularensis* are shown. (sur., water samples; sed., sediment samples).

During the three-year study we collected 341 water surface samples and 245 sediment samples in total. The *F. tularensis*-specific *lpnA* sequence was detected in 108 (32%) and 48 (20%) samples, respectively, using real-time PCR screening (Figure 2). The sequences were detected in samples obtained at several sampling points in both study regions and in each year.

### 3.4. Detailed Studies of Selected Sampling Points

**Figure 3:**
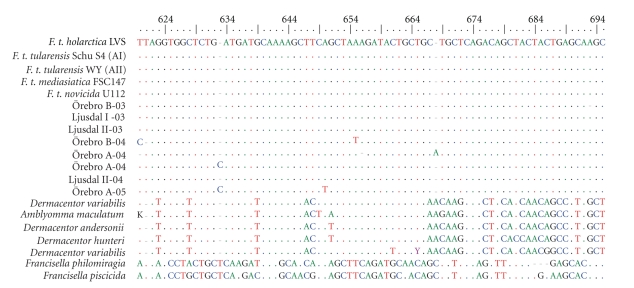
Multiple alignment of *lpnA* sequences obtained from Ljusdal and Örebro with previously published sequences of *Francisella *species and subspecies and *Francisella*-like endosymbionts (FLE). The nucleotide positions 620 to 695 refer to *F. t. holarctica* LVS (M32059). Reference sequences from GenBank: *F. t. holarctica* LVS (M32059), *F. t. tularensis* strain WY96-3418 (CP000608), *F. t. tularensis* strain Schu S4 (NC_006570), *F. t. mediasiatica *strain FSC147 (NC_010677), *F. t. novicida* strain U112 (CP000439), *Dermacentor variabilis *FLE (AY375420), *Amblyomma maculatum *FLE (AY375422), *Dermacentor andersonii* FLE (AY375413), *Dermacentor hunteri *FLE (AY375417), *Dermacentor variabilis *FLE (AY375421), *F. philomiragia* (AY243030) and *F. piscicida* (DQ825765).

Two sampling points from Ljusdal (I and II) and Örebro (A and B) that were consistently positive in the *lpnA* assay were retrospectively selected for detailed analysis (Figure 1). A total of 54 samples were analysed from these four locations over the three-year sampling period and 24 of the samples were *lpnA*-positive (Table 1), and it proved possible to sequence eight of these (Figure 3). The sequences were compared with published sequences from representatives of all described *F. tularensis* subspecies and their closest known relatives, and found to be 95%–100% similar (Figure 3).

**Figure 4:**
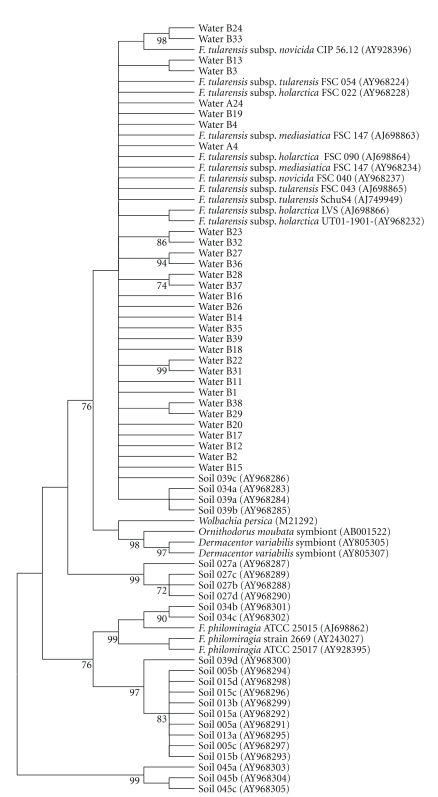
Phylogenetic analysis based on *Francisella* 16S rRNA sequences obtained from water samples in Örebro (Water A/B, this study), environmental soil samples [6] and reference sequences from GenBank. The samples are named according to the sampling points, Örebro A and B (Figure 1). Analysis was performed using maximum parsimony.

In order to further investigate the occurrence of *Francisella* DNA in water from the selected sampling points, we amplified 16S rRNA using the 16S rRNA primers for *Francisella*-like organisms reported by Barns et al. 2005 [6]. In total, 30 sequences were obtained, all of which grouped exclusively to the subspecies of species* F. tularensis* in the phylogenetic analysis (Figure 4).

**Figure 5:**
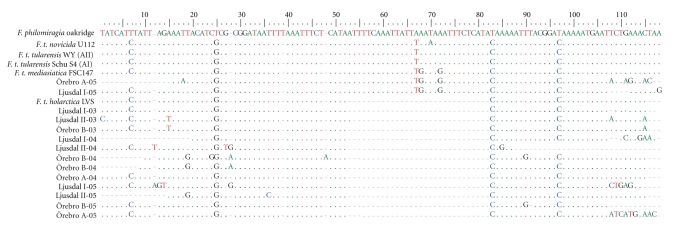
Multiple alignment of FtM19InDel sequences from Ljusdal and Örebro with previously published sequences of *Francisella *species and subspecies. In this alignment the *F. tularensis* subsp. *holarctica* specific deletion is located from position 52 to 82. Reference sequences from GenBank: *F. t. holarctica* LVS (M32059), *F. t. tularensis* strain WY96-3418 (CP000608), *F. t. tularensis* strain Schu S4 (NC_006570), *F. t. mediasiatica *strain FSC147 (NC_010677), *F. t. novicida* strain U112 (CP000439) and *F. philomiragia* (AY243030).

The *lpnA*-positive samples from the selected sampling points were subjected to FtM19InDel fragment size analysis, which has been shown to differentiate *F. tularensis *subsp. holarctica from other *F. tularensis *subspecies and *Francisella* -like bacteria. The fragments amplified corresponded to *F. tularensis *subsp*. holarctica* (100 bp) in 16 of the samples and to non-*holarctica Francisella*-like bacteria (130 bp) in two. Remaining 6 samples were negative presumably because the FtM19InDel primers are less sensitive than the *lpnA* primers. The sequences of the 100 bp amplicons (*n* = 12) showed high sequence similarities (95%–100%) to those of previously published *holarctica* strains (Figure 5 and Table 1). The 130 bp, full-size amplicons, (*n* = 2) aligned most closely with *F. tularensis *subsp. *mediasiatica* (Figure 5 and Table 1). *Francisella tularensis *subsp. *holarctica* sequences were detected in samples obtained at each of the four selected sampling points during all three years (Table 1).

## 4. Discussion

In this study, we used a molecular method to demonstrate the occurrence of the clinically relevant subspecies *F. tularensis* subsp. *holarctica* in water and sediment samples from two tularemia areas in Sweden, during three consecutive years. Water and sediment samples from the tularemia areas were screened for the presence of *F. tularensis* DNA using a PCR assay to amplify the *lpnA* gene [33]. This generates a product from all four *F. tularensis* subspecies, but not from other *Francisella* spp. or *Francisella*-like endosymbionts (FLE). Although not quantitative, the detection limit of the *lpnA* assay used here has been estimated to be 10^3^ bacteria per mL in natural water samples [27]. Therefore, the presence of PCR products from the water and sediment samples indicated the presence of *F. tularensis* in fairly high numbers. The *lpnA* assay, in contrast to previously performed animal inoculations [21], is potentially capable of detecting both pathogenic *F. tularensis* (i.e., subsp. *tularensis* and *holarctica*) and nonpathogenic *F. tularensis*. This might have contributed to the high frequency of *F. tularensis* in our samples over the three-year study period (108 positive out of 341 water samples analyzed). Since we initially expected low frequencies of *F. tularensis*-positive samples, we investigated a large number of sampling points during the first year of the study. However, due to the high detection rate, the number of sampling points was reduced in the following two years.

Sequence analysis of 16S rRNA clones amplified from *lpnA*-positive samples confirmed that the template organisms exclusively grouped with the subspecies within species *F. tularensis*. In previously reported environmental study by Barns et al. 2005 [6], in which essentially the same procedure was used, the targeted bacteria were found to consist of a mixture of distantly related *Francisella*-like bacteria, including *F. philomiragia*. This implies that the water environments from which we cloned 16S rRNA sequences, were more selective for *F. tularensis* subspecies than the soil and sediment samples analyzed by Barns et al. [6].

Although related strains *F. philomiragia *and *F. tularensis* subsp. *novicida* can be cultured directly from water [10], this is not currently true for the clinically significant subspecies of *F. tularensis*, *tularensis* and *holarctica.* Nevertheless, the presence of subspecies *holarctica* in water and sediments has been proven through the isolation of culturable bacteria from laboratory animals inoculated with water samples [21]. In order to identify *F. tularensis *subsp. *holarctica* in water samples we developed the FtM19InDel assay. We previously analyzed a total of 688 *F. tularensis* strains for this marker and found a 100% correlation between the 30-bp deletion and subspecies *holarctica* (unpublished results). Here, we amplified the *F. tularensis* subsp. *holarctica* sequence (Figure 5) in the samples selected for detailed analysis (i.e., those from four sampling points that yielded samples with consistently positive results in the initial screen using the *lpnA* assay, Table 1 and Figure 1). On the contrary, the causative agent of human tularemia in North America, *F. tularensis *subsp.* tularensis* (type A), was not detected in environmental samples during an ongoing outbreak in the active natural focus on Martha's Vineyard (MA, USA) [38]. These findings may reflect differences in the environmental stability between *F. tularensis* subsp. *tularensis* and *holarctica* strains possibly due to differing ecological niches and reservoirs for the two subspecies.

Using the FtM19InDel assay we also obtained full-length fragments corresponding to non-*holarctica F. tularensis *subspecies, in samples from both Ljusdal and Örebro (Figure 5 and Table 1). Surprisingly, the sequences of these full-length InDelFt-M19 fragments showed high similarity to that of *F. tularensis *subsp. *mediasiatica*. This subspecies occurs as rare human pathogens in Kazakhstan and Uzbekistan, and has virulence comparable to that of strains of *F. tularensis* subsp. *holarctica *[39]. However, all clinical isolates originating from the Örebro and Ljusdal regions that we have typed so far (*n* = 151), belonged without exception to the subsp. *holarctica* [31]. Therefore, it is highly unlikely that the *F. tularensis* subsp. *mediasiatica*-like sequences detected in this region were derived from a human pathogenic clone. Instead, this finding may reflect the diversity of *Francisella* and *F. tularensis*-like organisms in the environment, as evidenced by a growing body of data [6, 10].

Interestingly, we detected *F. tularensis* subsp. *holarctica* in water sampled in Ljusdal during 2004, when no human cases were recorded in the area. Thus, the presence of *F. tularensis* subsp. *holarctica* in water is not necessarily sufficient for spread of the disease to susceptible hosts. Occurrence of the bacterium in water during the nonoutbreak year suggests that, in addition to the bacterial contamination of water during ongoing outbreaks (from bacteriuria or decomposing carcasses) [16, 40, 41], *F. tularensis* subsp. *holarctica* persists in water between outbreaks. In a recent study, Svensson et al. (2009) combined epidemiologic investigations with high-resolution genotyping of *F. tularensis* subsp. *holarctica* isolates obtained from patients in the same regions, Örebro and Ljusdal [31]. In line with our results, Svensson et al. observed that genetic subpopulations of the bacteria were present throughout the tularemia season and persisted over years [31]. We also detected *F. tularensis* subsp. *holarctica* in water samples from the same sampling points during three consecutive years, indicating that the bacterium may persist in water for several years. The intervals between tularemia outbreaks often span several years, or even decades. Experience from rodent models and human outbreaks suggest that there is no healthy chronic carrier stage [42]. Thus, neither shedding nor carcass contamination can explain the bacterial persistence between outbreaks.

We included analysis of rodents to investigate a potential correlation between persistence of *F. tularensis* in water and rodents. In 2003, 97 rodents were live caught and investigated for the presence of the bacterium. Two of the rodents, both caught in Örebro at different sampling points, were positive by culturing. The obtained isolates were identified as two distinct* F. tularensis* subsp. *holarctica *strains and thus likely contracted from different sources. Due to a drop in rodent population sizes, only seven individuals were trapped in Örebro during 2004, despite extended number of trap nights as compared to 2003. All seven were *F. tularensis* negative. However, several water samples were positive for the presence of *F. tularensis* subspecies *holarctica* at the same sampling points. Taken together, *F. tularensis* subsp. *holarctica* can be found persistent in water also in the absence of infected rodents. Moreover, the results show that surveillance of *F. tularensis* in the environment using rodents as sentinels is not reliable over years and between outbreaks.

Laboratory experiments have shown that *F. tularensis* subsp. *holarctica* can survive in water for months [43]. However, within days after release in water, the bacterium enters a viable but nonculturable (VBNC) state [27, 43, 44]. Whole-genome sequencing has shown that *F. tularensis* subsp. *holarctica* has a low metabolic capacity, suggesting that it is an obligate host-dependent bacterium [45]. Further, *F. tularensis* subsp. *holarctica* shows enhanced survival when co-cultured with certain types of protozoa, indicating that ubiquitous protozoa might be an important environmental reservoir for the bacterium [26–29]. The aquatic systems sampled in this study (Örebro and Ljusdal tularemia areas) could be characterized as eutrophic systems [46]. In such systems with high nutrient availability, the bacterial population has been shown to be structured by protozoan predation pressure [46, 47]. In turn, mosquito larvae, mainly of the species *Aedes sticticus* and other flood-water mosquitoes, have been shown to exert a significant predatory impact on a protozoan population in a temporarily flooded wetland [30]. Altogether, this indicates that mosquito larvae may be exposed to *F. tularensis* subsp. *holarctica* in the water environments investigated here. Accordingly, we identified *F. tularensis *DNA in mosquitoes reared to adults in the laboratory, from larvae collected in temporary waters in the tularemia area (Örebro) [unpublished, Lundström et al. 2010]. Moreover, Svensson et al. 2009, identified an association between disease clusters (i.e., locations of tularemia transmission via mosquitoes) and recreational areas adjacent to water in the Ljusdal and Örebro tularemia areas [31]. As stated above, mosquito bites are the major route of transmission in both study regions [13] (Berglund L, personal communication).

The natural life-cycle of *F. tularensis* and the environmental reservoir of the bacteria have long been subject to speculation. Our working hypothesis is that *F tularensis* subsp. *holarctica *persists in water and/or sediment between tularemia outbreaks. Data presented here support this hypothesis, although the factors promoting the spread of the bacterium to susceptible hosts remain to be revealed.

## Figures and Tables

**Table 1:** Reported human tularemia cases (County Medical Officer, Gävle-Sandviken, personal communication, and Swedish Institute for Infectious Disease Control, SMI, Örebro County) and F. tularensis subsp. holarctica culture-positive rodents in the Ljusdal and Örebro areas during the study period. The total numbers of trapped rodents are shown in parentheses. Results of the molecular analysis of water samples from the four sampling points selected for detailed examination. Positive lpnA assay results indicate the presence of F. tularensis. The length of resulting FtM19InDel sequences indicates the presence of F. tularensis subsp. holarctica or other F. tularensis subspecies (other ssp.). n.d., not detected; n.s., not sampled.

			Ljusdal		Örebro	
			I	II	A	B
2003	Human cases		1		150	
	Rodents		0 (60)		2 (37)	
	Water					
		No. of samples tested	(4)	(4)	(2)	(2)
		*lpnA*	2	2	n.d.	1
		FtM19InDel	1	1		1
			*holarctica*	*holarctica*	n.d.	*holarctica*

2004	Human cases		0		54	
	Rodents		n.s.		0 (7)	
	Water					
		No. of samples tested	(7)	(7)	(8)	(8)
		*lpnA*	2	1	2	4
		FtM19InDel	2	1	2	3
			*holarctica*	*holarctica*	*holarctica*	*holarctica*

2005	Human cases		18		25	
	Rodents		n.s.		n.s.	
	Water					
		No. of samples tested	(3)	(3)	(3)	(3)
		*lpnA*	3	3	3	1
		FtM19InDel	1+1	2	1+1	1
			*holarctica*+ other* ssp. *	*holarctica*	*holarctica*+ other* ssp. *	*holarctica*
